# Estimation of Gait Parameters for Adults with Surface Electromyogram Based on Machine Learning Models

**DOI:** 10.3390/s24030734

**Published:** 2024-01-23

**Authors:** Shing-Hong Liu, Chi-En Ting, Jia-Jung Wang, Chun-Ju Chang, Wenxi Chen, Alok Kumar Sharma

**Affiliations:** 1 Department of Computer Science and Information Engineering, Chaoyang University of Technology, Taichung City 41349, Taiwan; shliu@cyut.edu.tw (S.-H.L.); s10827103@gm.cyut.edu.tw (C.-E.T.); 2 Department of Biomedical Engineering, I-Shou University, Kaohsiung 82445, Taiwan; 3 Department of Golden-Ager Industry Management, Chaoyang University of Technology, Taichung City 41349, Taiwan; cjchang@cyut.edu.tw; 4 Division of Information Systems, School of Computer Science and Engineering, The University of Aizu, Aizu-Wakamatsu City 965-8580, Fukushima, Japan; wenxi@u-aizu.ac.jp

**Keywords:** surface electromyogram, gait parameters, machine learning, decision tree, random forest, XGBoost, GaitUp Physilog^®^ wearable inertial sensors

## Abstract

Gait analysis has been studied over the last few decades as the best way to objectively assess the technical outcome of a procedure designed to improve gait. The treating physician can understand the type of gait problem, gain insight into the etiology, and find the best treatment with gait analysis. The gait parameters are the kinematics, including the temporal and spatial parameters, and lack the activity information of skeletal muscles. Thus, the gait analysis measures not only the three-dimensional temporal and spatial graphs of kinematics but also the surface electromyograms (sEMGs) of the lower limbs. Now, the shoe-worn GaitUp Physilog^®^ wearable inertial sensors can easily measure the gait parameters when subjects are walking on the general ground. However, it cannot measure muscle activity. The aim of this study is to measure the gait parameters using the sEMGs of the lower limbs. A self-made wireless device was used to measure the sEMGs from the vastus lateralis and gastrocnemius muscles of the left and right feet. Twenty young female subjects with a skeletal muscle index (SMI) below 5.7 kg/m^2^ were recruited for this study and examined by the InBody 270 instrument. Four parameters of sEMG were used to estimate 23 gait parameters. They were measured using the GaitUp Physilog^®^ wearable inertial sensors with three machine learning models, including random forest (RF), decision tree (DT), and XGBoost. The results show that 14 gait parameters could be well-estimated, and their correlation coefficients are above 0.800. This study signifies a step towards a more comprehensive analysis of gait with only sEMGs.

## 1. Introduction

Examination and comprehension of human movement have been enduring areas of scientific investigation. Gait parameters, which encompass intricate patterns and exhibit distinctive characteristics during the locomotion of walking and running, assume a pivotal position within a wide array of disciplines [1]. These disciplines span the domains of biomechanics and rehabilitation medicine, extending to sports science and human–computer interaction [2]. The precise prediction and assessment of these parameters have the potential to assess the identification of deviations from normal movement patterns in people with neurological diseases (like Parkinson’s), cardiopathies, sequelae from stroke, and diseases caused by aging and provide the treating physicians with the particular treatment programs [3,4,5]. Additionally, such predictions and assessments can contribute to the development of more productive assistive devices and the development of therapeutic interventions [6].

A surface electromyogram (sEMG) is used to record the electrical activity produced by muscle contraction, examine neuromuscular activity, and assist in the interpretation of kinematic and kinetic information [7]. In the intricacies between the neuromuscular system and motion, sEMG offers significant data pertaining to activation patterns, muscular strength, and the phenomenon of fatigue [8]. In the past, researchers have investigated the complex association between sEMG characteristics and gait measures using conventional statistical techniques, which have generally provided only partial or fragmented understanding [9,10]. Numerous studies have substantiated that the onset of sarcopenia can be discerned through the use of sEMG techniques or via the analysis of gait parameters [11]. However, it is pertinent to note that the examination of gait parameters primarily elucidates the repercussions of lower extremity muscle malfunction. It shows limited insights into the comprehensive gait effects precipitated by muscle dysfunction. In contrast, analyzing the lower extremity through sEMG predominantly offers data pertaining to the muscle activation status during dysfunction without offering substantial information on the consequential impacts on gait [12].

The traditional gait analysis uses charge-coupled device (CCD) cameras to capture the three-dimensional kinematic dynamics in temporal and spatial domains while a subject walks on a walkway [13,14]. sEMG is usually measured to determine whether the muscle is active or inactive during walking and to help in the evaluation of its dynamic power and condition. However, this system can only be used in a motion laboratory, and subjects cannot naturally walk on a general road. Agostini et al. proposed the problems for sEMG applied to gait analysis [9]. The limitations of sEMG are a lack of reference patterns and poor repeatability, reproducibility, and reliability [11]. Now, the shoe-worn GaitUp Physilog^®^ (Gait Up SA., CH-1015, Renens, Switzerland) wearable inertial sensors are a powerful alternative to traditional gait assessment in the motion laboratory [15,16]. Subjects wearing the shoe-worn sensors on the shoes can naturally walk on the general ground, and the temporal–spatial parameters of gaits are measured in real time.

Recent advances in machine learning (ML) have shown promising results in healthcare analytics, offering a more efficient approach for predicting and understanding complex medical conditions [17,18]. Moreover, ML models have been widely used in physiological measurements for estimating physiological parameters such as blood pressure [19,20], muscle mass [21], calories [22,23], glucose [24], and left ventricular ejection fraction [25]. In the classification applications, ML models performed the signal qualities of an electrocardiogram [26] and a photoplethysmogram [27,28], arrythmia detection [29], and risky activities in daily life [30]. When using an ML method to process the regression or classification problem, searching for the major features and finding the appropriate ML algorithms will depend on the collected data [31,32]. The processing of features is an important issue that can directly affect the performance of the ML model. The more accurate the features, the higher the performance of the ML model. Although some traditional statistical analysis methods have good results for clinical prediction in some cases, ML methods reignite interest in exploiting these fields [33,34].

As mentioned previously, the gait analysis not only includes the temporal and spatial parameters of the lower limbs but also needs the dynamic information of these muscles. Although shoe-worn wearable sensors measure the parameters of gaits, they cannot measure the electrical activities of muscles. A 3D motion camera system, although the most accurate, is bulky and costly. It is not compatible with daily life activities. Now, some textile integrations are replaced with surface electrodes for long-term bioelectronic potential measurement. Thus, the sEMG has the chance to be compatible with daily life activities.

Thus, the aim of this study is to explore the temporal and spatial parameters of gaits measured by sEMGs of the lower limbs with the ML methods. The sEMGs were measured from two major muscles of two feet, the vastus lateralis and the gastrocnemius muscles, and the commercial gait analysis system, GaitUp Physilog^®^ 5, measures the gait parameters as the reference. A wireless sEMG device was designed in this study to measure the sEMGs when subjects were running on the treadmill. Six features of sEMG were used to estimate 23 gait parameters by 3 ML models, namely decision tree (DT), random forest (RF), and XGBoost. Twenty female subjects participated in this study whose skeletal mass index (SMI) was below 5.7 kg/m^2^. The results showed that the performances of RF and XGBoost are almost equal. The Pearson correlation coefficients (PCCs) of fourteen gait parameters are above 0.800. The proposed method has the potential advantage of serving as a wearable gait analysis system with only sEMGs.

## 2. Materials and Methods

**Figure 1:**
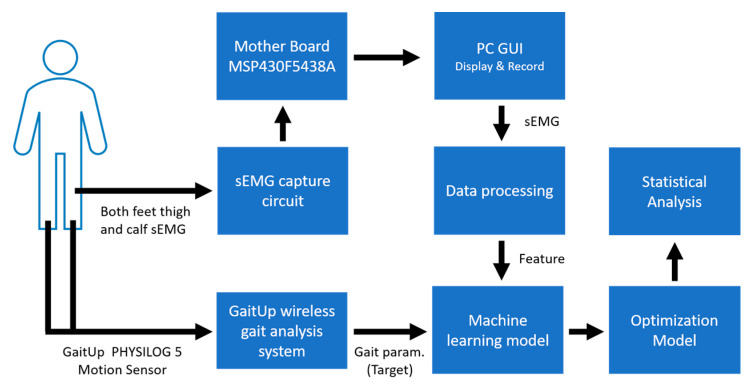
Overall system architecture diagram.

The layout of the proposed system is shown in Figure 1. The designed sEMG measurement system uses the XBee module (Digi International XBee^®^, Hopkins, MD, USA) to transmit four-channel sEMG signals to the personal computer (PC). The GaitUp Physilog^®^ 5 sensors are fixed on the shoes. Initially, the captured sEMG signals are processed through a digital filter to remove the noise as the raw sEMG (Raw_sEMG), followed by a one-order discrete wavelet transform to extract the high-frequency component (DWTH_sEMG). Then, six features are, respectively, extracted from the Raw_sEMG and DWTH_sEMG. The gait parameters are estimated by the ML models. Moreover, the models with better performances would be optimized to enhance their performances. Finally, the results are analyzed by the statistical evaluation methods.

### 2.1. Data Measurement Devices

The devices utilized in this research include a self-built sEMG apparatus and a pair of shoe-worn GaitUp Physilog^®^ wearable inertial sensors.

#### 2.1.1. GaitUp Physilog^®^ 5 System

**Figure 2:**
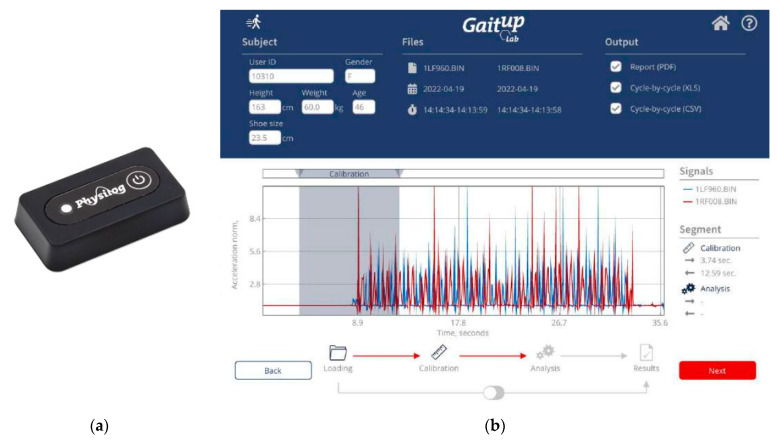
(**a**) The photo of the shoe-worn GaitUp Physilog^®^ 5 wearable inertial sensor and (**b**) the interface of the GaitUp Lab gait analysis system.

Figure 2 shows the GaitUp Physilog^®^ analysis tool, including the shoe-worn GaitUp, specifically the Physilog^®^ 5 wearable inertial sensor (Figure 2a), and the analysis software (Figure 2b) [35]. This supplemental tool aids in monitoring walking patterns by garnering precise quantitative data through pre- and post-gait evaluations. This facilitates medical practitioners and therapists in identifying areas of gait weakness and potential hazards associated with the subjects. Within the scope of this study, the GaitUp Lab gait analysis system was employed to measure the individual 23 gait parameters of the left and right feet, which include 9 temporal parameters, 9 spatial parameters, 4 ground clearance analyses, and a singular turn analysis. Table 1 shows the descriptions of 23 gait parameters.

#### 2.1.2. sEMG Measuring Device

**Figure 3:**
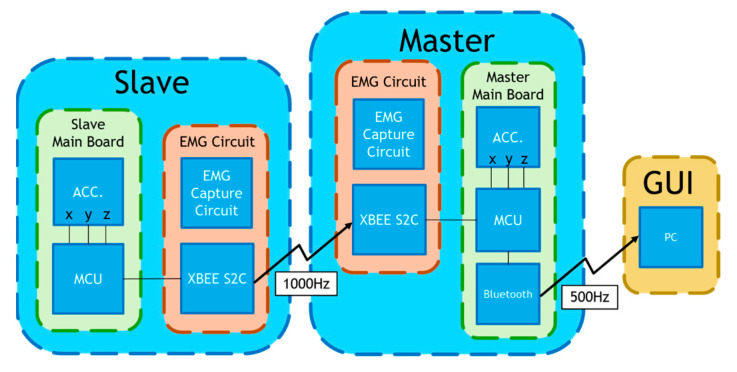
A self-made multi-channel wireless sEMG measurement device.

**Figure 4:**
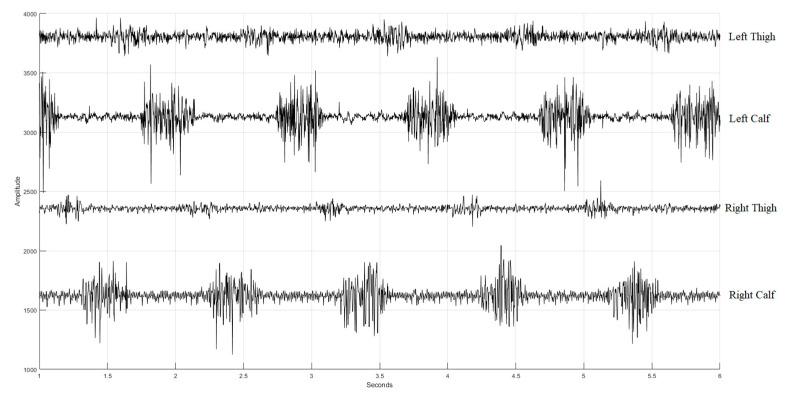
Four sEMG signals (with additive offsets in the vertical axis) during walking. The first row is the sEMG of the left thing, the second row is the sEMG of the left calf, the third row is the sEMG of the right thing, and the fourth row is the sEMG of the right calf.

We developed a multi-channel wireless sEMG monitoring device, as depicted in Figure 3. Two boards (slave and master) employ the XBEE S2C modules for data exchange between them with a transmission rate of 1000 bytes/s. Additionally, the master board incorporates the HC-05 Bluetooth module to forward the acquired sEMG data to a personal computer (PC) with a transmission rate of 500 bytes/s. The board has two channels of two sEMGs and an accelerometer (not used in this study). The sEMG circuit inherits the study of Liu et al. [36]. The device’s microcontroller is based on the Texas Instruments (TI) MSP430F5438A, whose analog-to-digital resolution (ADC) is 12 bits. Thus, the package transmitted to the PC terminal has 2 head bytes and 20 data bytes. In Figure 4, the time sequence of the four sEMG signals measured by the device during walking is presented, spanning a duration of 5 s. These sEMGs show the biomechanical movements associated with ambulation, explicitly focusing on the contracting strengths of the thigh and calf muscles for both the left and right lower extremities.

### 2.2. Data Processing

**Figure 5:**
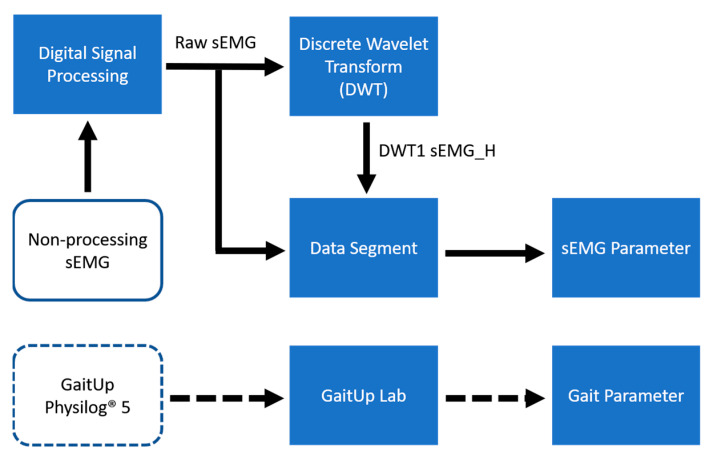
Data processing flowchart.

Figure 5 depicts a flowchart of the data processing architecture, illustrating the process of handling data in the study. Initially, the recoded sEMG data were subjected to digital filtering to eliminate noise, resulting in Raw_sEMG. Subsequently, the decomposition of DWT yielded DWTH_sEMG. Data segmentation was then performed on both the Raw_sEMG and DWTH_sEMG signals, followed by the calculation of time and frequency domain parameters. The data from shoe-worn GaitUp Physilog^®^ 5 wearable inertial sensors were segmented and processed via GaitUp Lab (Gait Up SA., CH-1015, Renens, Switzerland) software to extract gait parameters as the reference.

#### 2.2.1. Digital Filtering

A median filter was deployed to neutralize the spike disturbances originating from wireless communication packet discrepancies. The Raw_sEMG was then processed by a 4th-order Butterworth bandpass filter with a 20–240 Hz passband to remove the wandering baseline and high-frequency noise.

#### 2.2.2. Discrete Wavelet Transform

Existing studies highlighted that the high-frequency component of sEMG exhibited enhanced sensitivity for monitoring muscle fatigue under dynamic contraction [8]. The DWT is a widely utilized technique for processing data and is noted for its ability to separate high- and low-frequency components from a discrete signal [37]. Based on these findings, this study employed the primary DWT to extract the high-frequency component, DWTH_sEMG.

#### 2.2.3. Data Segmentation

**Figure 6:**
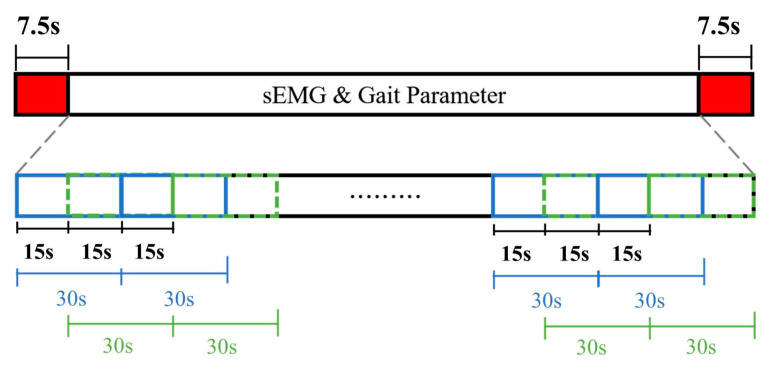
Data segmentation diagram.

The recorded data was 6 min in time, or 180,000 points for both Raw_sEMG and DWTH_sEMG. The initiation of data segmentation was aligned with the inception of intentional muscle contraction noted in the sEMG recordings, coupled with the commencement of activity noted within the GaitUp Lab, serving as the benchmark for the onset of data cutting. To mitigate the effects of gait variations at the experiment’s start and end, this study excluded 7.5 s from both the beginning and the end of the signal. This led to a total elimination of 15 s from the signal, resulting in a post-deletion signal length of 172,500 points. Regarding data segmentation, both the sEMGs and the shoe-worn GaitUp Physilog^®^ 5 wireless sensors were configured to segment the data into batches, each lasting 30 s, with a window shift of 15 s. This setup allowed for the extraction of 22 sample sets from every experiment, as shown in Figure 6. The data recorded by GaitUp Physilog^®^ 5 were segmented and processed using the GaitUp Lab software to yield gait parameters.

#### 2.2.4. sEMG Parameters

This study incorporated the common sEMG parameters, as defined in previous studies [38,39,40,41]. The mean frequency (MF) was derived from the product of the signal’s power spectrum and its frequency. This resultant value was then divided by the cumulative sum of the power spectrum, as shown in Equation (1):

(1) $$MF=\sum_{j=1}^{M}f_{j}p_{j}/\sum_{j=1}^{M}p_{j}$$

where $f_{j}$ represents the *j*th frequency of sEMG relative to the *j*th power spectrum, $p_{j}$, and *M* is the number of discrete frequency harmonics.

The median frequency (MDF) separates the signal’s power spectrum into two equal portions, as shown in Equation (2).

(2) $$\sum_{j=1}^{MDF}p_{j}=\sum_{j=MDF-1}^{M}p_{j}=\frac{1}{2}\sum_{j=1}^{M}p_{j}.$$

The standard deviation (STD) formula is shown in Equation (3).

(3) $$STD=\sqrt{\frac{1}{2}\sum_{i=1}^{N}{\left(x_{i}-\bar{x}\right)}^{2}},$$

where *x* is sampled data and *N* is the number of samples.

The root mean square (RMS) is given in Equation (4), and the average power (AP) in Equation (5).

(4) $$RMS=\sqrt{\frac{\sum_{i=1}^{n}x_{i}^{2}}{n}}.$$

(5) $$AP=\frac{1}{T}\sum_{j=1}^{T}x_{j}^{2},$$

Sample entropy (SampEn) is a parameter used to assess the regularity and complexity of a time series. A higher entropy value indicates increased complexity in the time series. A signal of length *N* is denoted as:

(6) $$X=\{x_{1},x_{2},x_{3},\ldots ,x_{N}\}.$$

Computing sample entropy necessitates specifying the embedding dimension *m* and the tolerance value *r*. Once the dimension *m* is established, the signal *X* can be divided into several segments, denoted by $X_{m}$:

(7) $$X_{m}\left(i\right)=\{x_{i},x_{i+1},x_{i+2},\ldots ,x_{i+m-1}\}.$$

Equation (8) represents the sample entropy.

(8) $$SampEn=-ln\frac{A}{B},$$

where

$A=d\left[X_{m+1}\left(i\right),\;X_{m+1}\left(j\right)\right]<r$,
$B=d\left[X_{m}\left(i\right),\;X_{m}\left(j\right)\right]<r$.
*d* […] indicates the Euclidean distance or Manhattan distance?

In this study, we set *m* = 2 and *r* = STD(sEMG) × 0.2. Thus, Raw_sEMG and DWTH_sEMG signals from both the thigh and calf were extracted, resulting in a total of 24 sEMG parameters for a foot.

### 2.3. Experiment Protocol

In the study, all participants were voluntary and selected from adults diagnosed with sarcopenia symptoms, possessing healthy limbs, and exhibiting standard standing postures. The cohort consisted of 20 female subjects aged from 19 to 23 years, with a mean ± standard deviation of 20 ± 1 years. The average height and weight were 156 ± 4.6 cm (ranging from 149 to 164 cm) and 45.9 ± 5.7 kg (spanning 31 to 56 kg), respectively. The muscle mass of the right foot was 4.95 ± 0.68 kg (ranging from 3.31 to 6.15 kg), the muscle mass of the left foot was 4.94 ± 0.68 kg (ranging from 3.24 to 6.03 kg), and the skeletal muscle index (SMI) was 5.07 ± 0.49 kg/m^2^ (ranging from 3.5 to 5.7 kg/m^2^). Furthermore, the subjects had an average shoe size of 23.9 ± 0.6, with sizes ranging from 23 to 25 cm. This experiment was approved by the Research Ethics Committee of Chung Shan University Hospital (No. CS2-22210) in Taichung City, Taiwan.

**Figure 7:**
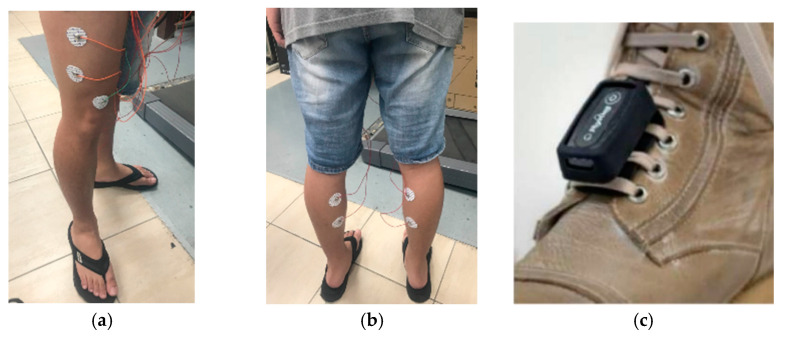
(**a**) The electrodes are attached to the vastus lateralis muscle, (**b**) the electrodes are adhered to the gastrocnemius muscle, and (**c**) the GaitUp sensor is tied to the shoe tongue.

In the beginning, the participants’ muscle masses in their feet and their SMI were measured using the InBody270 body composition analyzer (InBody Co. Ltd. Korea). The participants were recruited as subjects when their SMIs were below 5.7 kg/m^2^ for females and 7.0 kg/m^2^ for males [42]. The study employed a self-designed multi-channel wireless sEMG device for sEMG recordings in conjunction with the shoe-worn GaitUp Physilog^®^ 5 wearable inertial sensors to gather gait metrics. Prior to partaking in the experimental procedures, subjects were required to self-evaluate their overall physical health. This research applied the following procedures to gather data for the study:

1. In the sEMG measurement, there were four distinct sets of electrodes. These are individually adhered to the vastus lateralis and gastrocnemius muscle dips and plateau regions related to the presence of an innervation zone on each foot [11]. As shown in Figure 7, the reference electrode is positioned on the knee-facing side of the rectus femoris muscle (shown in Figure 7a,b). We avoided the belly position of the muscle and shifted the electrodes to a higher position. The surface electrodes used for the sEMG recording were Ag/AgCl with a 10 mm diameter on self-adhesive supports. The positions of the electrodes for each subject were recorded, and the electrodes were placed at the same position in the subsequent experiments. The shoe-worn GaitUp Physilog^®^ 5 wearable inertial sensors were placed on the tongue area of both shoes, as shown in Figure 7c. It was essential to ascertain that the electrodes and sensors remained secure, preventing any potential loosening from movement that could compromise data integrity.
2. The subjects ran on the treadmill at a speed of 5 km per hour for a duration of 6 min.
3. The subjects would evaluate their physical state and make necessary modifications during each experiment, all ensuring at least a 10 min break.
4. Subjects were measured four times. The interval between any two measurement sessions is at least a week.

### 2.4. Regression Model

In this study, the regression model training was facilitated using a system outfitted with an i7-10700 processor, an NVIDIA GeForce RTX3070 (NVIDIA, Santa Clara, CA, USA) graphics card, and 64 GB of memory at 2933 MHz. To predict the gait parameters, the study employed three prevalent nonlinear regression models, such as DT, RF, and XGBoost, all of which were developed by Python.

#### 2.4.1. Decision Tree

A decision tree (DT) [43,44] is a model characterized by a hierarchical tree framework. Each node within the tree signifies various choices, leading sequentially to anticipated outcomes. Due to their distinctive configuration, DTs are markedly clear and facilitate easy comprehension of the model’s predictive guidelines. Consequently, DT has emerged as one of the most renowned nonlinear regression models. DT is noted for its proficiency in nonlinear regression tasks, adeptly modeling intricate relationships between inputs and outputs without necessitating a linear correlation.

#### 2.4.2. Random Forest

The random forest tree (RF) [45] model is an advanced method of the conventional DT technique, leveraging the Random Subspace Learning algorithm to formulate trees characterized by low inter-correlations. Consequently, the RF [46] strategy amalgamates a multitude of low-correlation DTs, culminating in a substantial decision forest. Due to the enhanced randomness introduced within the model structure, it is observed that models conceived through the RF tree approach exhibit heightened robustness, thereby mitigating the propensity for overfitting, which underscores the efficacy of this methodology in complex predictive analytics.

#### 2.4.3. XGBoost

XGBoost (Extreme Gradient Boosting) [47] stands as an improved version of the Gradient Boosting technique. Bearing structural resemblances to a DT, it amalgamates numerous weak DTs to craft a potent predictive model. When compared with conventional classification and regression techniques, XGBoost typically exhibits superior accuracy [48]. Given its robust adaptive learning capabilities, XGBoost retains its prominence as a frequently employed classification and regression model in contemporary research and on competitive platforms.

### 2.5. Statistical Analysis

The quantitative data are expressed as the mean ± standard deviation. A computation of the PCC, *r*, is performed to establish the relationship between the predicted values and the target values found in the test data. This correlation coefficient is delineated in Equation (9). Here, $m_{x}$ represents the average of the x-vectors and $m_{y}$ denotes the average of the y-vectors.

(9) $$r=\frac{\sum \left(x-m_{x}\right)\left(y-m_{y}\right)}{\sqrt{\sum {\left(x-m_{x}\right)}^{2}\sum {\left(y-m_{y}\right)}^{2}}}$$

## 3. Results

Following the experiment, the total number of experiments executed by subjects was 68. The number of samples extracted from the sEMGs was 1496 for the left and right legs, respectively. Some samples missed the target output extracted by the GaitUp Lab software. There were 1477 viable samples for the left and right legs, respectively. A sample has 24 sEMG parameters: 12 parameters for Raw_sEMG and DWTH_sEMG of calf muscle and 12 for thing muscle. We methodically structured the samples into training and testing sets, adhering to a proportion of 8:2. Therefore, the numbers of training and testing samples were 1181 and 296, respectively. There were two models, left and right legs, for each gait parameter. Thus, the number of total models was 46.

Table 2 presents the statistical analysis of gait parameters for all samples, illustrating the minimum value, which indicates the lowest recorded point, and the maximum value, which signifies the highest point observed. Moreover, it also delineates the mean value, which is the average of all the collected data, along with the standard deviation, which showcases the extent of variation or dispersion of the set of values within the samples.

### 3.1. Selection of Gait Parameters

We hypothesized that the sEMG signals could not simply estimate all gait parameters. Thus, the XGBoost regression model was used to estimate the gait parameters of two feet. Table 3 shows the PCCs of all gait parameters. The PCCs between 0.7 and 1.0 (−0.7 and −1.0) indicate a strong positive (negative) linear relationship through the regression model [49]. Table 3 shows the correlation coefficients of all gait parameters. We defined two rules to determine what gait parameters could be estimated. The first rule was that the coefficients’ difference between two feet should not exceed 0.05. The second rule was that both the left and right feet must exhibit coefficients greater than 0.8 simultaneously. In Table 3, the gait parameters fitting these rules are marked with bold asterisks. There are six temporal parameters: gait cycle time, double-foot stance period, stride frequency, weight-bearing reaction period, mid-foot landing period, and propulsion period. Additionally, there are six spatial parameters: step length, stride length, maximum swing speed, toe-off ground angle, swing width, and three-dimensional walking length. The ground clearance analysis includes two parameters: the minimum toe clearance height and the maximum toe clearance height. In the initial phase of the study, the gait parameters requiring estimation are delineated.

### 3.2. Optimal sEMG Parameters

Normalizing feature importance can correct the bias in feature importance [50]. The method repeats permutations of the outcome vector to estimate the distribution of measured importance for each parameter. The *p*-value of the observed importance provides a corrected measure of parameter importance. In Table 4, we define the significance attributions associated with each sEMG parameter when it pertains to the efficacy of the established regression model. Remarkably, the statistical analysis reveals an absence of correlation, evidenced by a zero score, between the root mean square value and the average power. Thus, in the second phase, the MF, MDF, STD, and SampEn would be selected for the following assessment of the three ML models.

### 3.3. Regression Models

The DT, RF, and XGBoost were used to estimate the gait parameters. Table 5 shows the coefficients of 14 gait parameters for the left and right feet estimated by the three models. The RF model has the best performance, coefficients are 0.920 ± 0.016 and 0.916 ± 0.018 for left and right feet, respectively. The DT has the worst performance, and coefficients are 0.806 ± 0.033 and 0.774 ± 0.040 for left and right feet. The coefficients of XGBoost are 0.920 ± 0.016 and 0.916 ± 0.018 for the left and right feet, respectively. We find the performances of the RF and XGBoost models very close. Thus, the two models would be evaluated in the next phases.

### 3.4. Optimize Hyperparameters

In order to optimize DT, RF, and XGBoost models, the Optuna algorithm was used to fine-tune important hyperparameters [51]. The optimal hyperparameter configurations by Optuna and the respective range values established for these regression models are shown in Table 6.

### 3.5. Cross-Validation

In this phase, a five-fold cross-validation was used to examine the potential predictive bias presented by constant input samples during the model training phase [52]. This method facilitated the evaluation of the influences of varying training and test samples on the predictive outcomes generated by the trio of models under study. As explained in Table 7, the statistical analysis of the DT, RF, and XGBoost models shows the RF model demonstrating superior efficacy (0.915 ± 0.006), while the DT model lagged, exhibiting the worst performance (0.796 ± 0.017).

### 3.6. Training and Prediction Time

In this study, the efficiency of the optimized regression model was not only evaluated by the correlation coefficient but also considered both the training and prediction times, with the detailed findings showcased in Table 8. A notable observation is that the DT model exhibits a considerably shorter training time, 0.02 s, when compared with the RF and XGBoost models, 0.34 and 2.10 s, respectively. Furthermore, a closer time for the prediction underscores a distinct variance in performance across the models. Remarkably, both the RF and DT models manage to complete predictions in less than 0.01 s, presenting a favorable outlook in terms of speed and efficiency. In contrast, the XGBoost model lags behind, consuming a more substantial amount of time to finalize predictions compared to its counterparts.

### 3.7. Model Size

The other important point for this study is the memory space of the model because the proposed system will be deployed on an edge computing platform in the future. Table 9 shows the sizes of three models. A discernible difference is observed in the memory space occupied by each model. The empirical findings indicate that the DT model occupies a minimal memory space of 56 KB, which is considerably smaller than that required by the other two models. RF needs the most memory space, 11,045 kB, which is difficult to implement in the edge computing environment.

## 4. Discussion

The use of gait analysis in clinical practices began three decades ago. Inman et al. proposed the complete study of gait kinematics in 1981 [53]. Muro-de-la-Herran et al. explored a number of articles based on gait analysis methods and clinical applications from 2012 and 2013. The total number was 32; 40% of these articles were related to traditional CCD cameras systems, 37.5% presented inertial sensor-based systems, and the remaining 22.5% corresponded to other wearable sensor systems [5]. The number of nonwearable systems was less than that of wearable systems because the potential benefit of wearable systems would be that they can be operated in nonmedical environments, like the home and care center for the elderly. Now the wearable systems for the gait analysis are all off-line systems. The wearable devices only record the signals, and the parameters of gaits are calculated by their algorithms in the computer. Moreover, Liu et al. have proposed a sEMG patch [36]. In this study, we used the ML models to calculate these parameters. If the ML model is implemented in a microcontroller with the edge computing technique, the wearable system shows these parameters in real time. Therefore, the treating physicians can adjust the walking posture of the patient or modify the treatments.

The sEMG records muscle electrical activity, which has been identified as a potential biomarker for numerous neuromuscular disorders [54]. By capturing the subtle nuances in muscle activation and deactivation phases, sEMG provides a granular snapshot of muscle function. Moreover, the gait analysis belongs to the outcome of mechanic activity, which includes the temporal and spatial gait parameters and the information of joint kinematics measured by the accelerometer and gyroscope [55]. Thus, the gait analysis using the sEMG information will add value to the traditional gait analysis in clinical practice [56,57]. In this study, we successfully predicted the 14 gait parameters with the sEMGs of two feet using ML models. The best correlation coefficient approached 0.915 ± 0.006, which was regressed by the RF model. Thus, the sEMG signals could support both information about gait and muscle activity.

In this study, we focused on the collection and analysis of raw sEMG (Raw_sEMG) and DWT1 sEMG_H (DWTH_sEMG) signals from the thigh and calf of the left and right feet. Our efforts and methodologies allowed us to successfully derive four distinct sEMG parameters, MF, MDF, STD, and SampEn, for a single foot shown in Table 4. These parameters can potentially provide insights into muscle activity, function, and other relevant biomechanical aspects. But the RMS and AP parameters did not make any contribution. They all belong to the “energy” content of sEMG, which is hard to link to the muscle force, reflecting the degree of activation of skeletal muscles [58]. Thus, they did not have a relationship with the gait parameters.

**Figure 8:**
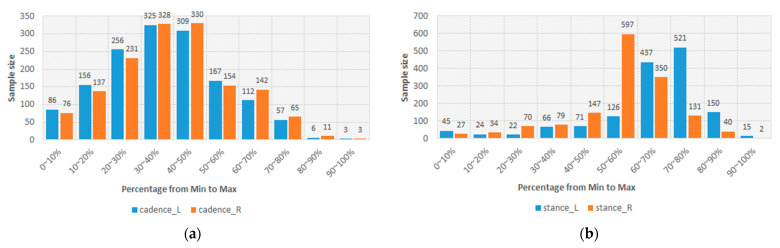
(**a**) The histograms of the “Cadence” parameter of the left and right feet and (**b**) the histograms of the “Stance” parameter of the left and right feet.

**Figure 9:**
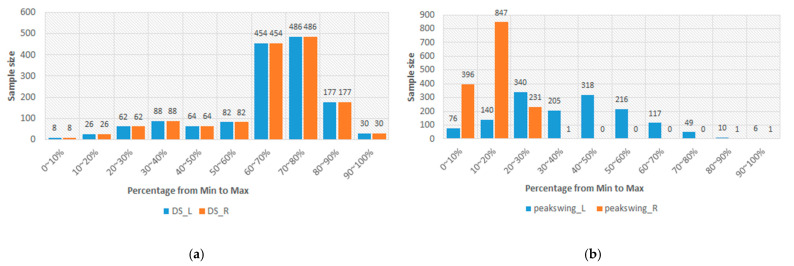
(**a**) The histograms of the “DS” parameter of the left and right feet and (**b**) the histograms of the “Peak Swing” parameter of the left and right feet.

**Figure 10:**
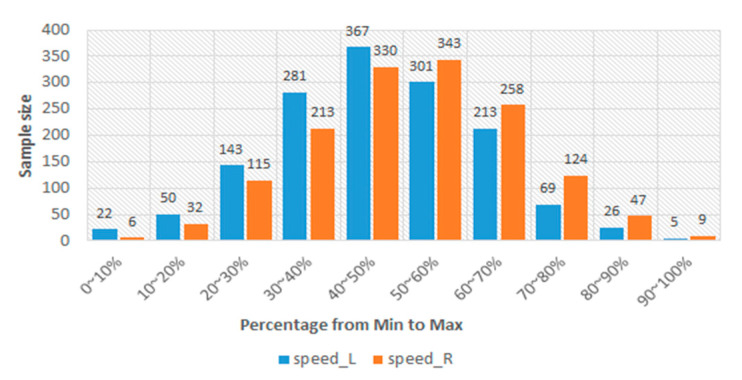
The histograms of the “Speed” parameter of the left and right feet.

In Table 3, the lowest PCC is 0.702. This result should represent that the sEMGs used to estimate the gait parameters are a potential research. Thus, the sEMGs have the potential benefit of approaching gait analysis with the ML technique. Moreover, we analyzed the reasons for the best and worst performances for estimating gait parameters according to the histograms of gait parameters. First, the more normal the distribution, the better the performance. Figure 8 shows the histograms of the “Cadence” and “Stance” parameters, whose coefficients are 0.913 and 0.909 vs. 0.917 and 0.780. The distribution of the “Stance” parameter is a disturbance, but for the “Cadence” parameter, it is the normal distribution. Second, the greater the difference between the gait-parameter distributions of left and right feet, the larger the difference between the estimating correlation coefficients. Figure 9 shows the histograms of the “DS” parameter and the “PAVS” parameter, whose coefficients are 0.931 and 0.893 vs. 0.883 and 0.793. The distributions of the left and right feet have a significant difference. Moreover, the palpability of the “PAVS” parameter of the right foot in the 10~20% range is significantly larger than the other ranges. Thus, the coefficient of the left foot is larger than the right foot, 0.883 vs. 0.793. However, the “Speed” parameter is an exception. Figure 10 shows the histograms of its left and right feet, which have normal distributions and are very close. But the coefficients of the “Speed” parameter are 0.776 and 0.798. The reason is that walking speed directly relates to the length and frequency of steps. The sEMG parameters do not have any length and frequency information. Thus, the “Speed” parameter could be difficult to estimate well.

This study not only compares the performance of RF, XGBoost, and DT models on several metrics, including PCC, training time, and prediction time, but also considers the model size. After combining all the results, this study observed that the RF model performed well in terms of PCCs for the individual gait parameters. However, in terms of model size, the RF model took up the most memory space, which shows that it may encounter limitations on lower configuration devices or edge computing environments. In the case of applying ML models to edge computing devices, the DT model may be more suitable, although its accuracy would need to be sacrificed. On the other hand, the XGBoost model achieved intermediate performance between the other two models in terms of correlation coefficient and model size. However, according to our analysis of the training and prediction times, it can be observed that the XGBoost model requires a longer training time, and its size is also larger, needing a memory space of 5805 kB, which is hardly enough to perform in edge computing settings.

The limitation of the current study pertains to the confined size of subject’s group, which restricts the broad applicability of our findings. Larger and more diverse samples in future studies could provide results that are both statistically more significant and more generalizable across different populations. Because the subject’s SMI should be below 5.7 kg/m^2^ for females and 7.0 kg/m^2^ for males, the gender of all subjects was female. This is also another limitation of this study. Although this study encompassed several ML models and sEMG parameters, the potential of unexplored or newly developed algorithms remains untapped. These could offer superior prediction accuracy and efficiency. Looking ahead, there’s a pressing need for future studies to incorporate a broader spectrum of sEMG feature mining and motion-sensing assistant. The final aim of this study will be to focus on the elderly population in the future because the elderly have a higher risk of dysfunctional walking. When elders have an abnormal walking pattern, the quality of their lives will decrease. Thus, the real-time gait information could allow the treating physicians to modify the treatment.

## 5. Conclusions

The study applied three ML models to effectively estimate the gait parameters using sEMGs measured from the lower limbs. These findings present a promising step towards more personalized and effective interventions for sarcopenia adults. Because the proposed method uses only sEMG signals to estimate the gait parameters, it has the potential to develop a wearable device based on edge computing in the future. If one combines MEC (mobile edge computing) with URLLC (ultrareliable low-latency communication) in 5G/6G, instantaneous data exchange and immediate patient feedback can be realized. Moreover, despite the progress made, future research should aim to expand the dataset and explore integration with other predictive technologies for more comprehensive and reliable gait analysis in elders with walking dysfunction.

## Figures and Tables

**Table 1:** The categories and abbreviations of 23 gait parameters.

Type	Gait Parameter	Name	Description
Temporal parameter	HS	Heel Strike	The point is when the heel hits the ground.
	CD	Cycle Duration	The time spent in one gait cycle is the time difference from the heel strike of the same foot to the next heel strike.
	DLS	Double Leg Support	The bipedal stance period is the proportion of the gait cycle when both feet are on the ground.
	Cadence	Cadence	Number of steps taken per minute.
	Stance	Stance Ratio	The stance period is the portion of the gait cycle during which the foot is in contact with the ground.
	SR	Swing Ratio	The swing period is the proportion of the gait cycle spent with the foot off the ground.
	LR	Load Ratio	The proportion of the stance period from the time when the heel strikes to when the sole of the foot touches the ground.
	FFR	Foot Flat Ratio	The proportion of the stance period in which the foot is completely flat on the ground.
	PR	Push Ratio	The proportion of the stance period from when the soles of the feet are flat to when the toes are off the ground.
Spatial parameters	Step Length	Step Length	The distance between one foot and the other foot on the ground.
	Strike Length	Stride Length	Distance from one heel strike to the next, equating one gait cycle.
	Speed	Gait Speed	Average forward walking speed.
	PAVS	Max. Angular Velocity During Swing	Maximum angular velocity in swing between the heel’s maximum height and the toe’s minimum height.
	SMTC	Foot Speed Norm at Minimal Toe Clearance	Maximum swing forward speed, typically over 3 times the pace, occurs near the minimum toe-off height.
	SA	Foot Pitch Angle at Heel Strike (Strike Angle)	The angle of the foot to the ground when it hits the ground.
	LoA	Foot Pitch Angle at Toe-Off (Lift-Off Angle)	Angle of toes to ground at the end of propulsion before lift-off.
	SW	Swing Width	The maximum lateral offset in the swing period equals the greatest lateral distance in the trajectory.
	Path Length	3D Path Length	Describes a 3D gait cycle trajectory scaled by stride length.
Turn analysis	TA	Turning Angle	Deflection angle for two consecutive flat steps of the same foot.
Ground clearance analysis	maxHC	Max. Heel Clearance	Heel reaches peak height off ground in each gait cycle.
	maxTC1	Max.1 Toe Clearance	After the heel’s peak height, the toes reach maximum height off the ground.
	minTC	Min. Toe Clearance	Minimum toe-off height during swing.
	maxTC2	Max.2 Toe Clearance	Toes reach maximum height off the ground before heels are ready to strike.

**Table 2:** The range, mean, and standard deviation of the two-foot gait parameters.

Parameter	Unit	MIN	MAX	AVE	STD
HS_L	Millisecond	9.03	15.83	14.76	0.71
HS_R	Millisecond	9.30	15.86	14.77	0.69
CD_L	Second	0.85	1.17	1.02	0.06
CD_R	Second	0.86	1.17	1.02	0.06
DS_L	% of cycle duration	8.05	26.96	20.35	3.25
DS_R	% of cycle duration	8.05	26.96	20.35	3.25
Cadence_L	steps/minute	103.00	141.18	117.50	6.67
Cadence_R	steps/minute	102.56	140.35	117.48	6.67
Stance_L	% of cycle duration	53.46	64.04	60.28	1.90
Stance_R	% of cycle duration	54.07	65.30	60.22	1.73
SR_L	% of gait cycle	35.96	46.54	39.72	1.90
SR_R	% of gait cycle	34.70	45.93	39.78	1.73
LR_L	% of stance	8.00	20.80	12.74	2.53
LR_R	% of stance	7.81	21.49	12.88	2.60
FFR_L	% of stance	33.77	56.03	47.32	4.67
FFR_R	% of stance	28.70	57.85	46.45	5.26
PR_L	% of stance	31.76	53.55	39.85	4.22
PR_R	% of stance	30.68	54.08	40.50	4.90
Step Length_L	Meter	0.53	0.74	0.64	0.04
Step Length_R	Meter	0.49	0.72	0.62	0.04
Strike Length_L	Meter	1.01	1.43	1.25	0.07
Strike Length_R	Meter	1.01	1.43	1.24	0.07
Speed_L	meter/s	1.18	1.28	1.23	0.02
Speed_R	meter/s	1.17	1.27	1.22	0.02
PAVS_L	degree/s	327.96	546.44	412.20	39.17
PAVS_R	degree/s	334.39	846.93	408.69	33.76
SMTC_L	meter/s	3.40	4.13	3.80	0.15
SMTC_R	meter/s	3.20	4.08	3.74	0.13
SA_L	Degree	10.35	34.33	23.02	4.79
SA_R	Degree	9.31	33.67	23.24	4.56
LoA_L	Degree	−97.11	−63.32	−81.29	7.11
LoA_R	Degree	−93.12	−66.14	−81.91	6.56
SW_L	Meter	0.01	0.08	0.04	0.02
SW_R	Meter	−0.09	−0.01	−0.05	0.01
Path Length_L	% of stride length	102.75	107.92	105.27	1.09
Path Length_R	% of stride length	102.78	109.14	105.34	1.04
TA_L	Degree	−1.61	1.70	0.21	0.50
TA_R	Degree	−2.44	1.14	−0.54	0.53
maxHC_L	Meter	0.22	0.36	0.26	0.02
maxHC_R	Meter	0.22	0.33	0.25	0.02
maxTC1_L	Meter	0.04	0.17	0.06	0.02
maxTC1_R	Meter	0.03	0.14	0.06	0.01
minTC_L	Meter	0.00	0.05	0.03	0.01
minTC_R	Meter	0.00	0.06	0.03	0.01
maxTC2_L	Meter	0.07	0.18	0.12	0.02
maxTC2_R	Meter	0.06	0.18	0.12	0.02

**Table 3:** Pearson correlation coefficients of gait parameters estimated by the XGBoost model.

Gait Parameter	Left Side	Right Side
HS	0.801	0.854
* CD	0.897	0.899
* DLS	0.931	0.893
* Cadence	0.913	0.909
Stance	0.917	0.780
SR	0.926	0.808
* LR	0.877	0.862
* FFR	0.917	0.906
* PR	0.907	0.907
* Step length	0.862	0.836
* Strike length	0.888	0.883
Speed	0.776	0.798
PAVS	0.883	0.793
* SMTC	0.919	0.913
SA	0.881	0.941
* LoA	0.932	0.913
* SW	0.903	0.874
* Path length	0.904	0.898
TA	0.748	0.723
maxHC	0.908	0.809
maxTC1	0.918	0.702
* minTC	0.851	0.812
* maxTC2	0.884	0.881

* The PCCs of both the left and right feet are greater than 0.8, and their difference is not greater than 0.05.

**Table 4:** Feature importance score.

Feature	Raw_sEMG		DWTH_sEMG	
	Thigh	Calf	Thigh	Calf
MF	0.08	0.17	0.07	0.05
MDF	0.11	0.07	0.02	0.03
STD	0.06	0.13	0.25	0.21
SampEn	0.11	0.14	0.09	0.23
RMS	0	0	0	0
AP	0	0	0	0

**Table 5:** Pearson correlation coefficients of 14 gait parameters for left and right feet estimated by DT, RF, and XGBoost models.

Name	Left Foot			Right Foot		
	DT	RF	XGBoost	DT	RF	XGBoost
CD	0.84	0.93	0.90	0.81	0.93	0.90
DLS	0.83	0.93	0.93	0.71	0.91	0.89
Cadence	0.84	0.93	0.91	0.80	0.93	0.91
LR	0.75	0.90	0.88	0.75	0.88	0.86
FFR	0.81	0.94	0.92	0.82	0.93	0.91
PR	0.78	0.92	0.91	0.84	0.93	0.91
Step length	0.80	0.91	0.86	0.76	0.91	0.84
Strike length	0.81	0.92	0.89	0.78	0.92	0.88
SMTC	0.83	0.94	0.92	0.81	0.93	0.91
SA	0.84	0.93	0.93	0.77	0.93	0.91
SW	0.82	0.93	0.90	0.76	0.91	0.87
Path length	0.78	0.91	0.90	0.79	0.93	0.90
minTC	0.74	0.88	0.85	0.74	0.88	0.81
maxTC2	0.82	0.92	0.88	0.70	0.91	0.88
Mean	0.806	0.920	0.899	0.774	0.916	0.884
STD	0.033	0.016	0.030	0.040	0.018	0.030

**Table 6:** Optuna’s Setting Values for hyperparameters of DT, RF, and XGBoost models.

Model	Hyperparameter	Range	Value
DT	max_depth	10~500	10
	min_simples_split	1~32	2
	min_samples_leaf	1~10	1
RF	n_estimators	100~500	50
	max_features	sqrt|log2	sqrt
	max_depth	5~15	15
	min_simples_split	1~32	2
	min_samples_leaf	1~10	1
XGBoost	n_estimators	500~100	200
	learning_rate	0.1~1.0	0.1
	max_depth	5~15	13
	subsample	0.1~1.0	0.8
	min_chilld_weight	0~300	30

**Table 7:** Five-fold cross-validation of RF and XGBoost models.

Models	Mean	STD
DT	0.796	0.017
RF	0.915	0.006
XGBoost	0.894	0.007

**Table 8:** Training and predicting times of DT, RF, and XGBoost.

Models	Training Time (s)	Predicting Time (s)
DT	0.02	≥0.01
RF	0.34	≥0.01
XGBoost	2.10	0.02

**Table 9:** The memory space occupied by the DT, RF, and XGBoost models.

Model	Size (KB)
DT	56
RF	11,045
XGBoost	5805

## Data Availability

Data are contained within the article.
